# Age and Self-Expansion Behaviors Correlate with Spatial Navigation in Healthy Adults

**DOI:** 10.3390/brainsci15091002

**Published:** 2025-09-16

**Authors:** Melissa Ansara, MaKayla Duggan, Alana Schafer, Karina Villalobos, Alexis N. Chargo, Ana M. Daugherty, Taylor N. Takla, Nora E. Fritz

**Affiliations:** 1 Neuroimaging and Neurorehabilitation Laboratory, Wayne State University, Detroit, MI 48201, USAmakdug@wayne.edu (M.D.);; 2 Department of Health Care Sciences, Wayne State University, Detroit, MI 48201, USA; 3 Institute of Gerontology, Wayne State University, Detroit, MI 48201, USA; alexis.chargo@wayne.edu (A.N.C.);; 4 Behavioral and Cognitive Neuroscience Program, Department of Psychology, Wayne State University, Detroit, MI 48201, USA; 5 Translational Neuroscience Program, Wayne State University, Detroit, MI 48201, USA; 6 Department of Neurology, Wayne State University, Detroit, MI 48201, USA

**Keywords:** spatial navigation, self-expansion, navigation performance, virtual reality, aging, motivation

## Abstract

**Background:** Spatial navigation is one’s ability to travel through their environment to reach a goal location. Self-expansion is the motivation to increase one’s self-perception through engaging in novel activities. Our objective was to examine the relations among self-expansion, age, and navigation ability and investigate how one’s internal motivation may influence navigation performance across paradigms. **Methods:** In total, 33 younger adults (YAs; 19F, 14M, mean age = 25.0 ± 1.6) and 74 older adults (OAs; 52F, 22M, mean age = 69.5 ± 8.0) completed the following: Self-Expansion Preference Scale (SEPS), Wayfinding Questionnaire (WQ), Virtual Supermarket Task, Virtual Morris Water Maze (vMWM), and a Floor Maze Task (FMT). Mann–Whitney U tests and Spearman ρ correlations were used to examine differences in navigation performance between YAs vs. OAs and self-expanders vs. self-conservers, and relations among the measures, respectively. **Results:** YAs had lower vMWM completion times compared to OAs (*p* < 0.001). Self-expanders had better recall of the vMWM environment compared to self-conservers (*p* = 0.049), independent of age. Greater self-expansion in YAs was correlated with lower spatial anxiety (*ρ* = −0.356, *p* = 0.042) and faster completion of the FMT (*ρ* = −0.36, *p* = 0.042). **Discussion:** Our results build on established age-related deficits in navigation abilities to identify correlations of self-expansion and better performance in various navigation tasks. Independent of age, individuals with greater inclination towards self-expansion exhibit superior navigation abilities. Future research should explore underlying mechanisms driving these associations and investigate intervention strategies aimed at improving navigation skills in aging populations through increasing self-expansion.

## 1. Introduction

Spatial navigation (SN) requires complex cognitive processing to support individuals in reaching goal locations efficiently and accurately [1]. SN abilities, including knowledge about landmarks, travel routes, and integration of sensory and spatial information are necessary for efficient navigation and vulnerable to aging [2,3,4,5]. Age-related decline in navigation, like remembering route details, are particularly apparent in novel environments [5], and are associated with increased risk for neurodegenerative diseases like dementia [6]. SN decline can negatively impact independence, travel abilities, and overall safety for older adults (OAs) [7], making this a topic warranting further investigation.

While cognitive and functional correlates of age-related differences in SN have been identified [7,8], the role of intrinsic motivational factors in exploratory behaviors across ages has not been closely examined. Personality traits related to openness and anxiety have been correlated with navigation efficiency and accuracy [9]. It is plausible that individuals who pursue novel experiences may exhibit greater proficiency in everyday navigation. Self-expansion describes the preference to seek new experiences, gain new resources, and contribute to personal achievement [10]. Self-expansion is associated with increased engagement in novel experiences, greater self-efficacy, improved psychological well-being, and healthier habits [11,12]. As successful navigation is essential for independent living and community engagement, understanding associations of self-expansion with navigation performance may inform strategies to encourage active aging and mitigate negative declines in navigation performance.

To better understand these concepts, SN can be evaluated with immersive virtual reality (IVR), non-immersive virtual reality (NI-VR), and overground navigation tasks. Virtual reality (VR) has emerged as a valuable research tool in assessing SN in controlled, simulated environments [13]. Both IVR and NI-VR have been used to evaluate cognitive processes that are relevant for successful navigation, such as working memory and route learning [14,15]. IVR provides a sense of physical presence, with strong correlations of proprioceptive and visual cues in the simulated space [16]. NI-VR training can enhance problem solving, attention, and memory in OAs [17]. Moreover, research suggests that IVR and NI-VR yield comparable results in simple wayfinding tasks such as landmark identification to reach a destination [18].

There is evidence to suggest greater willingness to explore surroundings promotes navigation efficiency [19]. Therefore, the purpose of our study was to examine the relations of age, self-expansion behaviors, and SN abilities in IVR, NI-VR and overground tasks. We aimed to assess whether younger adults (YAs) perform better on navigation tasks compared to OAs in novel environments and whether individuals who frequently engage in novel experiences perform better on SN tasks. We hypothesized that YAs would outperform OAs in virtual environments and those with a greater tendency towards self-expansion behaviors would demonstrate better performance across ages.

## 2. Materials and Methods

### 2.1. Participants

YAs were recruited from the metro-Detroit area via mass email invitation, and performed a digital pre-screening survey to ensure that they met the following inclusion criteria: aged 18–30, ambulatory without an assistive device, and able to follow study-related commands. Participants were excluded from the study if they had a history of a neurological or psychiatric disease, a diagnosis of a vestibular disorder, or an acute orthopedic injury that affected ambulation.

OAs were part of a larger parent study, recruited from the longitudinal Detroit Aging Brain Study, and met the following inclusion criteria: ≥50 years old, had a high school education or equivalent, and native English-speakers. Exclusion criteria included presence of significant eye disease with accompanying visual impairment, depressive symptoms as indicated by ≥16 on the Center for Epidemiologic Studies Depression Inventory (CES-D), <27 on the Mini-Mental State Examination (MMSE), history of a head injury with loss of consciousness exceeding 5 min, presence of neurological, cardiovascular, psychiatric, or mobility-related disorder, drug or alcohol abuse, cognitive impairment, history of a learning disability, corrected visual acuity less than 20/60, hearing poorer than 40 dB for 1000, 2000, 3000, and 4000 Hz frequencies, and use of centrally acting prescription drugs (i.e., tranquilizers and analgesics).

All study procedures were approved by the Wayne State University Institutional Review Board, and eligible participants provided informed e-consent prior to participation. Survey measures were collected using REDCap, a HIPAA compliant data capture tool prior to the study visit. All objective SN tasks were collected in a single in-person laboratory visit. YAs completed self-reported measures (demographics, Self-Expansion Preference Scale [SEPS], Wayfinding Questionnaire [WQ]) and laboratory SN assessments (IVR Supermarket Task, NI-VR Virtual Morris Water Maze [vMWM], and Floor Maze Task [FMT]). OAs completed demographic reports, the vMWM, and the SEPS (Table 1). It is important to note that YA and OA participants were collected as separate samples and were merged for the purpose of this study.

### 2.2. Self-Reported Questionnaires

#### 2.2.1. Self-Expansion Preference Scale (SEPS)

The SEPS is a validated 24-item scale that measures one’s inclination towards self-expansion and self-conservation behaviors [11]. Self-expansion is the motivation to engage in novel activities, whereas self-conservation describes one’s preference to participate in routine and familiar experiences [11]. Participants used a 7-point ordinal scale to answer each item [11]. Higher scores on the SEPS indicate greater self-expansive preferences. The final question required participants to select from two available options (i.e., forced choice) what they felt best described them: self-expander or self-conserver.

#### 2.2.2. Wayfinding Questionnaire (WQ)

The WQ is a validated 22-item questionnaire that assesses individuals’ self-perceived navigation abilities [20]. Participants answered each item using a 7-point ordinal scale, and outcomes included 3 sub scores: navigational orientation, spatial anxiety, and distance estimation. Higher sub scores in each category indicate greater self-perceived orientation abilities, more anxiety about navigating unfamiliar places, and better distance estimation, respectively.

### 2.3. Immersive Virtual Reality

The MotionVR+ (Virtualis VR, Montpellier, France) is an IVR system equipped with a head-mounted display and hand-held controllers.

#### The Supermarket Task (Figure 1A)

The Supermarket Task is a module within MotionVR+ that measures SN and memory in an immersive virtual supermarket. Participants used the head-mounted display and hand-held controllers to “walk” through the virtual space. Individuals were given 5 min to explore the supermarket and familiarize themselves with the controllers. After 5 min, participants were given 20 s to memorize a shopping list of 5 standardized items. Individuals were then placed back in the supermarket to collect the items in no particular order. Navigation performance was recorded using the total time to complete the task.

**Figure 1:**
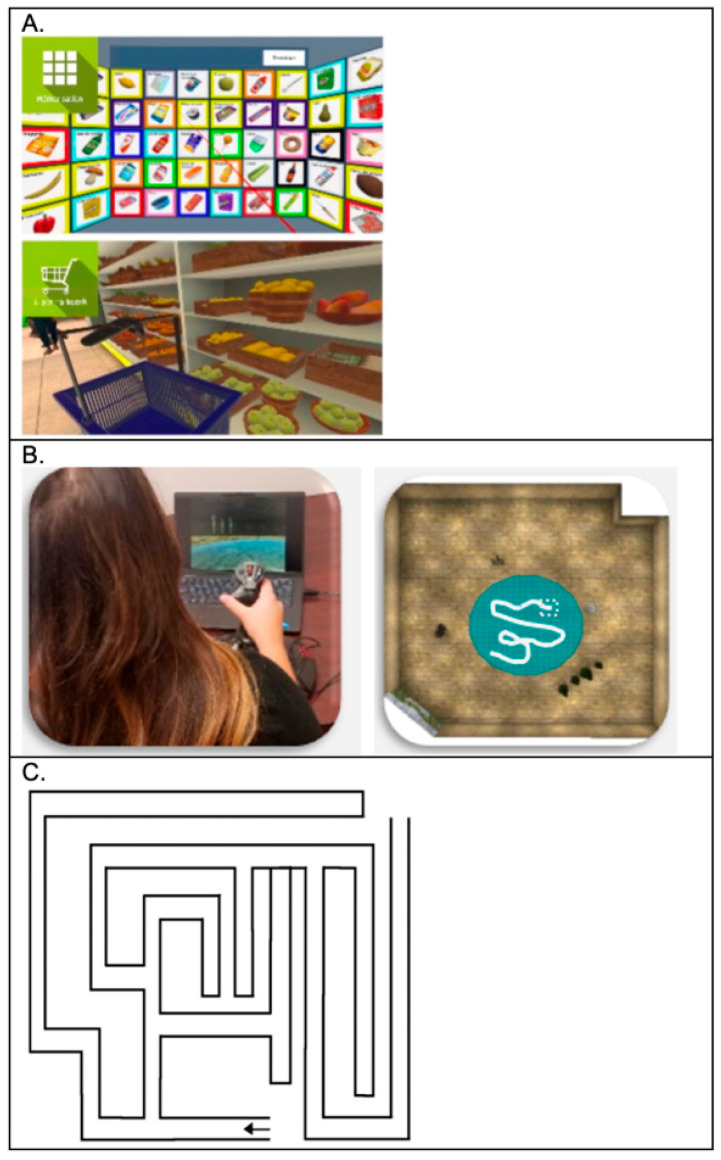
Spatial Navigation Assessments; (**A**) IVR Supermarket task view from headset; (**B**) vMWM task with first person perspective and overhead view; and (**C**) Overhead view of overground FMT. The arrow indicates the starting location of the maze, IVR: Immersive Virtual Reality; vMWM: virtual Morris Water Maze; FMT: Floor Maze Task.

### 2.4. Non-Immersive Virtual Reality (NI-VR)

#### Virtual Morris Water Maze (vMWM) (Figure 1B)

The vMWM task is a well-validated virtual laboratory assessment of navigation that was modeled after the traditional Morris Water Maze [8,21,22,23]. Before testing, participants completed a practice trial to familiarize themselves with the virtual interface and joystick control to navigate over five visible platforms, labeled A–E. The virtual test environment featured a circular pool within a larger room surrounded by several landmarks and two unique wall features. Participants were instructed to locate a hidden platform as quickly as possible. Participants completed 5 learning trials with a stationary platform location. Each trial was initiated from one of five unique starting positions in the three quadrants without the platform, equidistant from the platform, with facing direction selected at random. Trials were limited to two minutes and automatically terminated if the platform was not found within that time. Navigation efficiency was measured as the amount of time (seconds) and distance (virtual units) traveled from the start to the platform. Averages for time and distance were calculated across all learning trials where the participant successfully reached the platform. Average time and distance were included in data analysis only if the participant successfully completed 3 or more trials. An incomplete trial could result from a participant not finding the platform within the 2 min time limit, or prematurely ending the trial.

Environment Recall. After completing the vMWM, participants were provided with a blank sheet of paper and instructed to draw an overhead diagram of the vMWM room as a measure of environment recall. Participants were told to include as many details as possible of the room and pool, and to mark the location of the hidden platform with an “X”. Recall was scored for number of landmarks correctly depicted and accurately placed, relative to the marked platform location.

### 2.5. Overground SN

#### Floor Maze Task (FMT) (Figure 1C)

The FMT is an overground task used to assess navigation ability and executive functioning. Participants completed the maze twice: an initial trial and a delayed recall trial. In the initial trial, participants walked through a 7 × 10-foot floor maze as quickly as possible. The measures recorded during this trial included *planning time* (time from end of verbal instructions to when first step is taken) and *immediate time* (time of first step taken to maze finish). After the initial trial, participants performed unrelated tasks for approximately 10 min before returning to the maze to perform the second trial. *Delayed recall* (total time spent in the maze during the second attempt) was recorded.

### 2.6. Statistical Methods

All statistical analyses were performed in IBM SPSS Statistics version 29.0 for Windows (IBM Corp., Armonk, NY, USA). Mann–Whitney U tests were conducted to compare performance on the vMWM between YAs and OAs, and between self-expanders and self-conservers. Spearman correlations were used to examine correlations between self-expansion scores with measures of SN across the full sample (combined YAs and OAs). Significance across all analyses was evaluated at *α* = 0.05. Significant results were further evaluated with linear regression to determine R^2^ values.

## 3. Results

Overall, 107 individuals participated in this study, including 33 YAs (19 females, mean (SD) age = 25.0 (1.6) and 74 OAs (52 females, mean (SD) age = 69.5 (8.0) (Table 2). Of the 107 participants, 94 completed the SEPS (33 YAs and 61 OAs), 104 completed vMWM environment recall (33 YAs and 71 OAs), and 87 participants’ vMWM average time and distance were calculated (30 YAs and 57 OAs).

### 3.1. OAs Demonstrated Worse Efficiency as Compared to YAs in the vMWM

**Figure 2:**
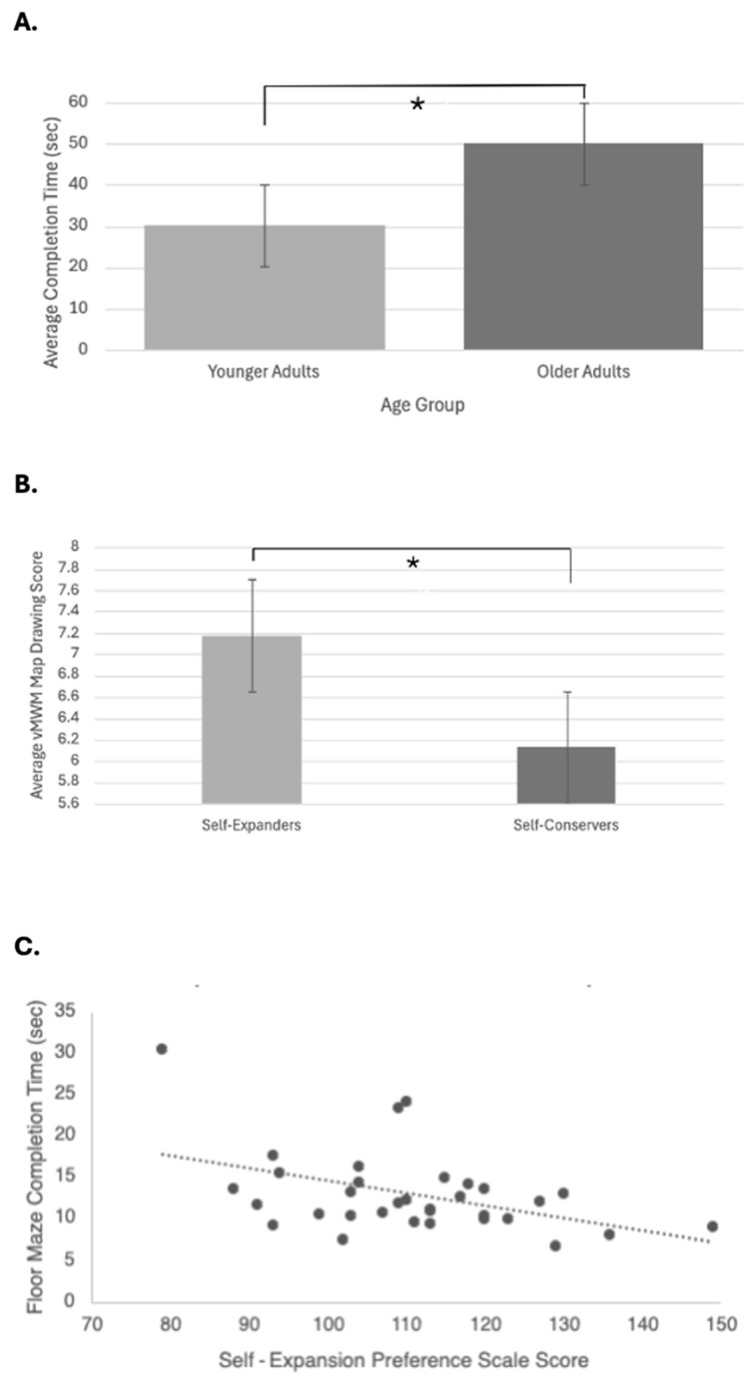
Older adults and self-conservers perform worse on SN tasks. (**A**) Average completion time of the vMWM trials in young adults vs. older adults. Average time for young adults was 30.17 sec (mean age 24.93), while average time for older adults was 50.11 sec (mean age 69.46); *p* < 0.001. (**B**) Average vMWM environment recall scores in forced choice self-expanders vs. self-conservers. Average score for self-expanders (*n* = 68) was 7.18, while average score for self-conservers (*n* = 23) was 6.13; *p* = 0.049. (**C**) Higher sum scores on the Self-Expansion Preference Scale were significantly correlated with faster immediate completion time on the floor maze task (*ρ* = −0.356, *p* = 0.042). vMWM: virtual Morris Water Maze. * indicates significance at *p* = 0.05.

Data for performance on the vMWM environment recall was collected from 71 OAs and 33 YAs, and average time and distance were calculated for 57 OAs and 30 YAs. YAs took less time to locate the hidden platform compared to OAs (*U* = 274.000, *Z* = −5.188, *p* < 0.001) (Figure 2A). There was no significant difference between age groups on environment recall (*U* = 1070.000, *Z* = −0.716, *p* = 0.474) or average distance traveled (*U* = 704.000, *Z* = −1.348, *p* = 0.178). No significant sex differences were found in whole group and within group analyses (Supplementary Tables S2–S4).

### 3.2. Self-Expansion Was Associated with Better Environment Recall

**Figure 3:**
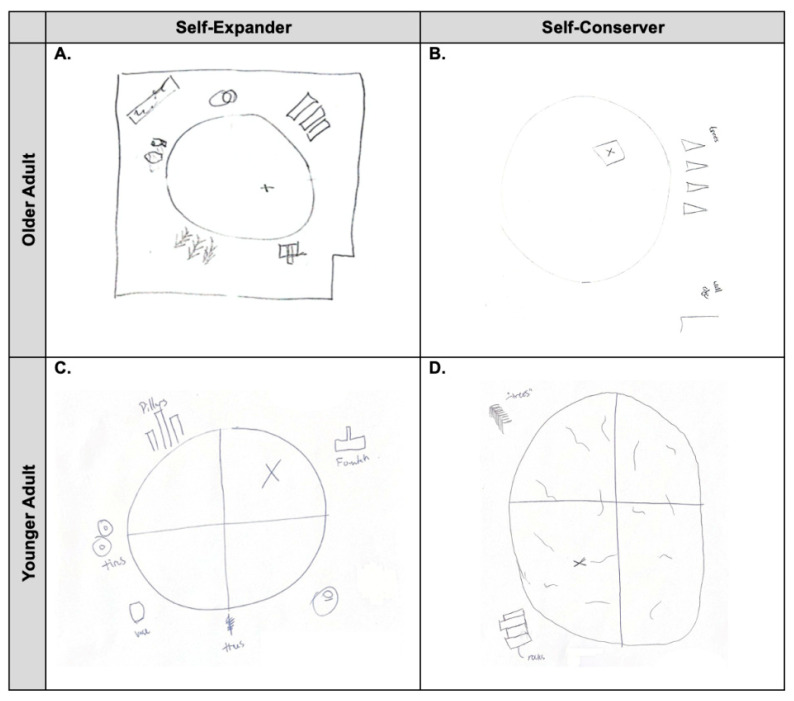
vMWM environment recall scores from four representative participants. (**A**,**B**) are older adults while (**C**,**D**) are younger adults; (**A**,**C**) are self-expanders while (**B**,**D**) are self-conservers. Differences between self-expanders and self-conservers can be recognized visually, aligning with our finding that higher map recall score correlates with better cognitive recollection of the virtual environment. vMWM: virtual Morris Water Maze.

Sixty-one OAs and 33 YAs completed the SEPS, with 68% of OAs and 61% of YAs self-selecting self-expander and 15% of OAs and 39% of YAs considering themselves self-conservers (Table 2). Self-expanders demonstrated higher scores on environment recall than self-conservers regardless of age, indicating better memory of environment details (*U* = 568.500, *Z* = −1.968, *p* = 0.049) (Figure 2B). No significant differences were found between groups in average vMWM completion time (*U* = 452.000, *Z* = −1.174, *p* = 0.240) or distance traveled (*U* = 439.000, *Z* = −1.330, *p* = 0.184). In this regard, self-expansion appears to encourage increased recollection of the environment, but a minimal number of details from the environment were required to efficiently navigate. Figure 3 highlights the differences between these groups with environment recall.

### 3.3. Self-Expansion Correlated with Better SN Performance Across Paradigms in YAs

The additional tasks and assessments completed by the YAs allowed a closer examination of the role of self-expansion. Greater self-expansion was associated with lower spatial anxiety (*ρ* = −0.409, *p* = 0.018; R^2^ = 0.110) and faster completion of the FMT (*ρ* = −0.356, *p* = 0.042; R^2^ = 0.201) (Figure 2C). No other significant correlations were found between self-expansion and remaining measures (Supplementary Table S1).

## 4. Discussion

This study investigated relations between self-expansion and SN performance between YAs and OAs across IVR, NI-VR, and overground environments. In the vMWM, YAs took less time to locate the hidden platform compared to OAs, supporting our hypothesis and results of previous studies that report less efficient and accurate navigation with advanced aging during real-world [2], overground [24], and virtual maze tasks [21,22,23,25]. Furthermore, previous research has indicated a significant difference in performance when comparing NI-IVR to IVR [26]. With NI-IVR environments, OAs can only rely on visual input; hindering an accurate representation of real-world SN [27]. In contrast, IVR SN can incorporate some degree or proprioceptive and vestibular input to conjure a more naturalistic performance [27]. Additionally, a recent systematic review and meta-analysis concluded that OAs exhibit poorer navigational performance and spatial learning than YAs in various navigation tasks, with greater differences between virtual environments and real-world tasks [28].

Our research demonstrates that self-expanders performed better on the vMWM environment recall task compared to self-conservers across ages. This finding supports our hypothesis that individuals who frequently seek novel experiences would perform better on SN tasks, and builds on existing literature to suggest that motivation towards self-expansive behaviors promotes better navigational strategies in novel environments [2,7,29]. Self-expanders may have greater cognitive adaptability, which is beneficial when navigating unfamiliar environments and encoding spatial information [10,11,12]. More attentive and active engagement along with cognitive flexibility may promote more effective use of environmental cues for spatial recall. Our findings add novel insight to documented age-related declines in navigational performance by integrating age and personality-driven motivation into a single framework. This study shows that self-expansion plays a role in environmental recall regardless of age, suggesting that motivational-based interventions might serve as an effective avenue for enhancing navigational abilities across the lifespan.

Among YAs, higher self-expansion sum scores were associated with faster completion of the immediate FMT, suggesting a link between self-expansion and better navigation performance in overground tasks. Interestingly, FMT performance has been shown to differentiate healthy OAs and those with cognitive decline [6,29]. Additionally, higher self-expansion sum scores were negatively correlated with WQ spatial anxiety, indicating that participants with an inclination to explore novel environments experienced less anxiety while navigating. This supports findings that personality plays a role in SN [9]. If an individual has heightened anxiety, they may take longer to complete the task if they are experiencing stress. Lower levels of spatial anxiety may facilitate more efficient navigation [30,31], allowing for individuals to allocate cognitive resources toward landmark details and environmental cues during route learning and wayfinding^21^. Our findings suggest that a willingness to engage in new experiences may augment navigation performance in real-world and NI-VR navigation tasks and correspond with reduced anxiety in novel environments.

While our hypotheses were partially supported, other expected relations were not statistically significant. Specifically, we found no correlation between self-expansion sum scores and navigation efficiency measures on the vMWM or supermarket tasks. Self-expanders have a greater capacity to be creative [32], meaning that when navigating through a new environment, they may not be more efficient in timing, but more resilient to change. In the vMWM, landmark cues are relevant for accurate navigation, but the platform can be found efficiently by selecting a few salient cues. Considering this, the correlation of self-expansion with greater environment recall and not navigation efficiency, suggests tendencies towards exploration may provide more detailed cognitive representation. Additionally, the SEPS asks about an individual’s preferences in real-world scenarios, which may not translate to performance in a simulated environment.

Some non-significant trends also emerged. A negative correlation between vMWM average time and WQ distance estimation suggests that individuals with stronger perceived distance estimation skills tended to complete the vMWM task faster (Supplementary Table S1). Additionally, a positive correlation between vMWM and IVR supermarket task completion times was observed, indicating that participants who navigated efficiently in NI-VR also performed better in IVR (Supplementary Table S1). This may be indicative of navigation performance across simulated environments, meaning IVR and NI-VR may be complimentary means of assessing task performance. However, correlations with IVR may not be fully reliable due to technical errors that occurred while using IVR. Lastly, greater spatial anxiety on the WQ was associated with worse FMT delayed recall time (not significant; Supplementary Table S1). Lack of correlation on performance of SN tasks with WQ in YAs may indicate that YA perception of their wayfinding abilities may not correlate to physical performance. Prior work highlights the lack of predictive value that wayfinding questionnaires have on SN performance in OAs, and that direct measures of wayfinding should be used over self-report questionnaires [33]. Additionally, mental imagery spatial anxiety may have been a contributing factor to increased FMT recall time, however mental imagery is not assessed in the WQ subset.

## 5. Limitations

While this study provides valuable insight on the interplay among SN, age, and self-expansion, there are several limitations to address. We acknowledge that our study did not include middle-aged adults (30–49 years), as this study aimed to evaluate differences between YAs and OAs. Importantly, YAs were largely students from the same graduate program at Wayne State University, indicating potential biases in terms of shared experiences or prior knowledge. During the use of IVR, some participants experienced technical difficulties (i.e., lagging and freezing of the virtual environment). Future research should consider utilizing an IVR that is reliable, valid, and technologically sound to prevent errors that may influence subject performance. Some participants experienced nausea during the VR tasks which may have impacted their current and subsequent task performance; data from these participants was excluded from analysis. Future studies should examine the role of vestibular dysfunction on spatial navigation performance. Finally, it is important to consider that OAs in general are less familiar with the technology used (see Table 2 for prior gaming and computer experience between groups), which could have contributed to their increased navigation time independent of age-related cognitive decline.

While self-expanders outperformed self-conservers on the vMWM environment recall task, no other significant differences were found on other vMWM measures between these two populations. Future studies should utilize more trials to better assess NI-VR navigation and incorporate overground tasks for OAs to provide a more environmental-based SN abilities unrelated to technology use. Lastly, future research should explore clinical relevance of self-expansion in physical activity and health outcomes, as existing literature has established that self-expansion positively correlates with physical activity [34]. Understanding the potential of self-expansion tendencies as an intervention could provide important implications for healthcare professionals seeking to enhance mobility, navigation, independence, and well-being in aging populations.

## 6. Conclusions

This study provides insight into relations between age, self-expansion behaviors, and SN performance in IVR, NI-VR, and overground environments. In line with prior work highlighting age-related decline in SN, our findings show that YAs consistently outperformed OAs on timed SN tasks in NI-VR. Additionally, our results suggest that regardless of age, individuals with higher self-expansion tendencies exhibit better SN performance, particularly in environmental recall. Finally, in YAs, a greater inclination toward self-expansion was associated with faster completion time on an overground navigation task (FMT). Together, these results highlight potential impacts of intrinsic motivation to explore novel environments on navigation abilities in various settings and reinforce the role of aging in navigation performance. Encouraging self-expansion through various means could assist in mitigating age-related decline in navigation abilities. Such interventions could provide practical applications for not only improving navigation abilities but also independence, self-efficacy, and quality of life, particularly in OAs.

## Figures and Tables

**Table 1:** List of measures by age group.

Tests	Older Adults (OA)	Younger Adults (YA)
Self-Expansion Preference Scale	X	X
Wayfinding Questionnaire		X
IVR Supermarket Task		X
Virtual Morris Water Maze	X	X
Floor Maze		X

**Table 2:** Participant Demographics.

	Older Adult (OA) (*n* = 74)	Younger Adults (YA) (*n* = 33)
Age (years), Mean ± SD	69.5 ± 8.0	25.0 ± 1.6
Sex, *n* (%)		
Female	52 (70.3%)	19 (57.6%)
Male	22 (29.7%)	14 (42.4%)
Race, *n* (%)		
Asian	1 (1.4%)	2 (6.1%)
Black	13 (17.6%)	0 (0.0%)
Middle Eastern	0 (0.0%)	3 (9.1%)
White	60 (81.1%)	27 (81.8%)
Other	0 (0.0%)	1 (3.0%)
Forced Choice on Self-Expansion Preference Scale, *n* (%)		
Self-Expander	50 (67.6%)	20 (60.6%)
Self-Conserver	11 (14.9%)	13 (39.4%)
Not Completed	13 (17.6%)	0 (0.0%)
Prior Computer Experience, *n* (%)		
Almost everyday	53 (71.6%)	33 (100.0%)
A few times per week	14 (18.9%)	0 (0.0%)
A few times per month	3 (4.1%)	0 (0.0%)
Several times per year	3 (4.1%)	0 (0.0%)
A few times per year	0 (0.0%)	0 (0.0%)
A few times in my life	1 (1.4%)	0 (0.0%)
I have never used a computer before	0 (0.0%)	0 (0.0%)
Prior Computer Game Experience, *n* (%)		
Almost everyday	6 (8.1%)	1 (3.0%)
A few times per week	2 (2.7%)	7 (21.2%)
A few times per month	5 (67.6%)	4 (12.1%)
Several times per year	0 (0.0%)	5 (15.2%)
A few times per year	10 (13.5%)	3 (9.1%)
A few times in my life	39 (52.7%)	13 (39.4%)
I have never played computer games	12 (16.2%)	0 (0.0%)
Prior 3D Environment Computer Game Experience, *n* (%)		
Almost everyday	0 (0.0%)	1 (3.0%)
A few times per week	0 (0.0%)	1 (3.0%)
A few times per month	0 (0.0%)	1 (3.0%)
Several times per year	1 (1.4%)	3 (9.1%)
A few times per year	5 (6.8%)	3 (9.1%)
A few times in my life	39 (52.7%)	17 (51.5%)
I have never played these games	29 (39.2%)	7 (21.2%)

Note: Includes sample size of each group (OAs and YAs), age with mean age presented as mean ± standard deviation, sex, race, forced choice self-expander or self-conserver concluded from participants self-identification of their willingness to participate in novel tasks, and prior computer and video game experience.

## Data Availability

The data presented in this study are available on request from the corresponding author due to reasons of privacy.

## Supplementary Materials

The following supporting information can be downloaded at https://www.mdpi.com/article/10.3390/brainsci15091002/s1, Table S1: Correlation matrix demonstrating relations of all included variables; Table S2: Whole group sex differences. 1= male, 2 = female; Table S3: Sex differences within OA population. 1 = male, 2 = female; Table S4: Sex differences within YA population. 1 = male, 2 = female.
