# Supplementary material for: Age and Self-Expansion Behaviors Correlate with Spatial Navigation in Healthy Adults
Source: Brain Sci. 2025 Sep 16;15(9):1002. doi: 10.3390/brainsci15091002 (PMC12468926; doi:10.3390/brainsci15091002)
Supplement: Supplementary file 1 [file brainsci-15-01002-s001.zip › brainsci-3834060-supplementary.pdf]

**Supplementary Table S1:** Correlation matrix demonstrating relations of all included variables

**Correlations**

| | | | Self-Expansion Sum Score | Wayfinding Questionnaire: Navigational Orientation | Wayfinding Questionnaire: Distance Estimation | Wayfinding Questionnaire: Spatial Anxiety | Supermarket Task: Grocery Store Time | Virtual Morris Water Maze: Environment Recall | Virtual Morris Water Maze: Average Time | Virtual Morris Water Maze: Average Distance | Floor Maze Task: Planning Time | Floor Maze Task: Immediate Time | Floor Maze Task: Delayed Recall |
|---|---|---|---|---|---|---|---|---|---|---|---|---|---|
| Spearman's rho | Self-Expansion Sum Score | Correlation Coefficient | 1.000 | .088 | .250 | **-.409**$^{*}$ | -.281 | .098 | .032 | .086 | -.149 | **-.356**$^{*}$ | -.199 |
| | | Sig. (2-tailed) | . | .628 | .160 | .018 | .126 | .354 | .785 | .464 | .408 | .042 | .267 |
| | | N | 94 | 33 | 33 | 33 | 31 | 91 | 75 | 75 | 33 | 33 | 33 |
| | Wayfinding Questionnaire: Navigational Orientation | Correlation Coefficient | .088 | 1.000 | **.709**$^{**}$ | **-.417**$^{*}$ | -.290 | .163 | -.294 | -.219 | -.254 | -.216 | -.308 |
| | | Sig. (2-tailed) | .628 | . | <.001 | .016 | .114 | .366 | .114 | .246 | .154 | .226 | .081 |
| | | N | 33 | 33 | 33 | 33 | 31 | 33 | 30 | 30 | 33 | 33 | 33 |
| | Wayfinding Questionnaire: Distance Estimation | Correlation Coefficient | .250 | **.709**$^{**}$ | 1.000 | **-.518**$^{**}$ | -.284 | .163 | -.360 | -.303 | -.090 | -.155 | -.139 |
| | | Sig. (2-tailed) | .160 | <.001 | . | .002 | .121 | .364 | .050 | .104 | .617 | .390 | .441 |
| | | N | 33 | 33 | 33 | 33 | 31 | 33 | 30 | 30 | 33 | 33 | 33 |
| | Wayfinding Questionnaire: Spatial Anxiety | Correlation Coefficient | **-.409**$^{*}$ | **-.417**$^{*}$ | **-.518**$^{**}$ | 1.000 | .208 | -.030 | -.096 | -.145 | .025 | .207 | .312 |
| | | Sig. (2-tailed) | .018 | .016 | .002 | . | .263 | .867 | .614 | .445 | .891 | .247 | .077 |
| | | N | 33 | 33 | 33 | 33 | 31 | 33 | 30 | 30 | 33 | 33 | 33 |
| | Supermarket Task: Grocery Store Time | Correlation Coefficient | -.281 | -.290 | -.284 | .208 | 1.000 | -.023 | .338 | .325 | .254 | .240 | .159 |
| | | Sig. (2-tailed) | .126 | .114 | .121 | .263 | . | .903 | .078 | .091 | .169 | .194 | .391 |
| | | N | 31 | 31 | 31 | 31 | 31 | 31 | 28 | 28 | 31 | 31 | 31 |
| | Virtual Morris Water Maze: Environment Recall | Correlation Coefficient | .098 | .163 | .163 | -.030 | -.023 | 1.000 | -.146 | -.136 | -.238 | -.251 | -.126 |
| | | Sig. (2-tailed) | .354 | .366 | .364 | .867 | .903 | . | .180 | .212 | .182 | .159 | .483 |
| | | N | 91 | 33 | 33 | 33 | 31 | 104 | 86 | 86 | 33 | 33 | 33 |
| | Virtual Morris Water Maze: Average Time | Correlation Coefficient | .032 | -.294 | -.360 | -.096 | .338 | -.146 | 1.000 | **.571**$^{**}$ | .149 | .062 | .118 |
| | | Sig. (2-tailed) | .785 | .114 | .050 | .614 | .078 | .180 | . | <.001 | .431 | .745 | .533 |
| | | N | 75 | 30 | 30 | 30 | 28 | 86 | 87 | 87 | 30 | 30 | 30 |
| | Virtual Morris Water Maze: Average Distance | Correlation Coefficient | .086 | -.219 | -.303 | -.145 | .325 | -.136 | **.571**$^{**}$ | 1.000 | .002 | .293 | .192 |
| | | Sig. (2-tailed) | .464 | .246 | .104 | .445 | .091 | .212 | <.001 | . | .990 | .117 | .309 |
| | | N | 75 | 30 | 30 | 30 | 28 | 86 | 87 | 87 | 30 | 30 | 30 |
| | Floor Maze Task: Planning Time | Correlation Coefficient | -.149 | -.254 | -.090 | .025 | .254 | -.238 | .149 | .002 | 1.000 | -.282 | .050 |
| | | Sig. (2-tailed) | .408 | .154 | .617 | .891 | .169 | .182 | .431 | .990 | . | .112 | .782 |
| | | N | 33 | 33 | 33 | 33 | 31 | 33 | 30 | 30 | 33 | 33 | 33 |
| | Floor Maze Task: Immediate Time | Correlation Coefficient | **-.356**$^{*}$ | -.216 | -.155 | .207 | .240 | -.251 | .062 | .293 | -.282 | 1.000 | **.563**$^{**}$ |
| | | Sig. (2-tailed) | .042 | .226 | .390 | .247 | .194 | .159 | .745 | .117 | .112 | . | <.001 |
| | | N | 33 | 33 | 33 | 33 | 31 | 33 | 30 | 30 | 33 | 33 | 33 |
| | Floor Maze Task: Delayed Recall | Correlation Coefficient | -.199 | -.308 | -.139 | .312 | .159 | -.126 | .118 | .192 | .050 | **.563**$^{**}$ | 1.000 |
| | | Sig. (2-tailed) | .267 | .081 | .441 | .077 | .391 | .483 | .533 | .309 | .782 | <.001 | . |
| | | N | 33 | 33 | 33 | 33 | 31 | 33 | 30 | 30 | 33 | 33 | 33 |

*. Correlation is significant at the 0.05 level (2-tailed).

**. Correlation is significant at the 0.01 level (2-tailed).

**Supplementary Table S2:** Whole group sex differences. 1= male, 2 = female.

**Ranks**

| | sex | N | Mean Rank | Sum of Ranks |
|---|---|---|---|---|
| Self-Expansion Sum Score | 1 | 32 | 45.08 | 1442.50 |
| | 2 | 62 | 48.75 | 3022.50 |
| | Total | 94 | | |
| Virtual Morris Water Maze: Environment Recall | 1 | 35 | 51.46 | 1801.00 |
| | 2 | 69 | 53.03 | 3659.00 |
| | Total | 104 | | |
| Virtual Morris Water Maze: Average Time | 1 | 31 | 39.32 | 1219.00 |
| | 2 | 56 | 46.59 | 2609.00 |
| | Total | 87 | | |
| Virtual Morris Water Maze: Average Distance | 1 | 31 | 40.68 | 1261.00 |
| | 2 | 56 | 45.84 | 2567.00 |
| | Total | 87 | | |

**Test Statistics**

| | Self-Expansion Sum Score | Virtual Morris Water Maze: Environment Recall | Virtual Morris Water Maze: Average Time | Virtual Morris Water Maze: Average Distance |
|---|---|---|---|---|
| Mann-Whitney U | 914.500 | 1171.000 | 723.000 | 765.000 |
| Wilcoxon W | 1442.500 | 1801.000 | 1219.000 | 1261.000 |
| Z | -.619 | -.254 | -1.285 | -.913 |
| Asymp. Sig. (2-tailed) | .536 | .800 | .199 | .361 |

**Supplementary Table S3:** Sex differences within OA population. 1 = male, 2 = female.

**Ranks**

| | sex | N | Mean Rank | Sum of Ranks |
|---|---|---|---|---|
| Self-Expansion Sum Score | 1 | 18 | 30.11 | 542.00 |
| | 2 | 43 | 31.37 | 1349.00 |
| | Total | 61 | | |
| Virtual Morris Water Maze: Environment Recall | 1 | 21 | 37.64 | 790.50 |
| | 2 | 50 | 35.31 | 1765.50 |
| | Total | 71 | | |
| Virtual Morris Water Maze: Average Time | 1 | 18 | 29.33 | 528.00 |
| | 2 | 39 | 28.85 | 1125.00 |
| | Total | 57 | | |
| Virtual Morris Water Maze: Average Distance | 1 | 18 | 26.78 | 482.00 |
| | 2 | 39 | 30.03 | 1171.00 |
| | Total | 57 | | |

**Test Statistics**

| | Self-Expansion Sum Score | Virtual Morris Water Maze: Environment Recall | Virtual Morris Water Maze: Average Time | Virtual Morris Water Maze: Average Distance |
|---|---|---|---|---|
| Mann-Whitney U | 371.000 | 490.500 | 345.000 | 311.000 |
| Wilcoxon W | 542.000 | 1765.500 | 1125.000 | 482.000 |
| Z | -.253 | -.439 | -.103 | -.687 |
| Asymp. Sig. (2-tailed) | .800 | .661 | .918 | .492 |

**Supplementary Table S4:** Sex differences within YA population. 1 = male, 2 = female.

**Ranks**

| | sex | N | Mean Rank | Sum of Ranks |
|---|---|---|---|---|
| Self-Expansion Sum Score | 1 | 14 | 16.04 | 224.50 |
| | 2 | 19 | 17.71 | 336.50 |
| | Total | 33 | | |
| Virtual Morris Water Maze: Environment Recall | 1 | 14 | 15.11 | 211.50 |
| | 2 | 19 | 18.39 | 349.50 |
| | Total | 33 | | |
| Virtual Morris Water Maze: Average Time | 1 | 13 | 13.46 | 175.00 |
| | 2 | 17 | 17.06 | 290.00 |
| | Total | 30 | | |
| Virtual Morris Water Maze: Average Distance | 1 | 13 | 14.85 | 193.00 |
| | 2 | 17 | 16.00 | 272.00 |
| | Total | 30 | | |
| Wayfinding Questionnaire: Navigational Orientation | 1 | 14 | 22.39 | 313.50 |
| | 2 | 19 | 13.03 | 247.50 |
| | Total | 33 | | |
| Wayfinding Questionnaire: Distance Estimation | 1 | 14 | 22.43 | 314.00 |
| | 2 | 19 | 13.00 | 247.00 |
| | Total | 33 | | |
| Wayfinding Questionnaire: Spatial Anxiety | 1 | 14 | 13.39 | 187.50 |
| | 2 | 19 | 19.66 | 373.50 |
| | Total | 33 | | |
| Supermarket Task: Grocery Store Time | 1 | 14 | 14.29 | 200.00 |
| | 2 | 17 | 17.41 | 296.00 |
| | Total | 31 | | |
| Floor Maze Task: Planning Time | 1 | 14 | 16.86 | 236.00 |
| | 2 | 19 | 17.11 | 325.00 |
| | Total | 33 | | |
| Floor Maze Task: Immediate Time | 1 | 14 | 18.79 | 263.00 |
| | 2 | 19 | 15.68 | 298.00 |
| | Total | 33 | | |
| Floor Maze Task: Delayed Recall | 1 | 14 | 16.71 | 234.00 |
| | 2 | 19 | 17.21 | 327.00 |
| | Total | 33 | | |

**Test Statistics**

| | Self-Expansion Sum Score | Virtual Morris Water Maze: Environmental Recall | Virtual Morris Water Maze: Average Time | Virtual Morris Water Maze: Average Distance | Wayfinding Questionnaire: Navigational Orientation | Wayfinding Questionnaire: Distance Estimation | Wayfinding Questionnaire: Spatial Anxiety | Supermarket Task: Grocery Store Time | Floor Maze Task: Planning Time | Floor Maze Task: Immediate Time | Floor Maze Task: Delayed Recall |
|---|---|---|---|---|---|---|---|---|---|---|---|
| Mann-Whitney U | 119.500 | 106.500 | 84.000 | 102.000 | 57.500 | 57.000 | 82.500 | 95.000 | 131.000 | 108.000 | 129.000 |
| Wilcoxon W | 224.500 | 211.500 | 175.000 | 193.000 | 247.500 | 247.000 | 187.500 | 200.000 | 236.000 | 298.000 | 234.000 |
| Z | -.492 | -.977 | -1.109 | -.356 | -2.753 | -2.782 | -1.842 | -.953 | -.073 | -.911 | -.146 |
| Asymp. Sig. (2-tailed) | .623 | .329 | .267 | .722 | .006 | .005 | .065 | .341 | .942 | .362 | .884 |
| Exact Sig. [2*(1-tailed Sig.)] | .627 | .339 | .281 | .742 | **.005*** | **.005*** | .065 | .356 | .957 | .377 | .900 |

*. Correlation is significant at the 0.05 level (2-tailed).
